# Sequence Analysis of the *K13*-Propeller Gene in Artemisinin Challenging *Plasmodium falciparum* Isolates from Malaria Endemic Areas of Odisha, India: A Molecular Surveillance Study

**DOI:** 10.1155/2020/8475246

**Published:** 2020-03-15

**Authors:** Ramakanta Rana, Manoranjan Ranjit, Madhusmita Bal, Hemant Kumar Khuntia, Sanghamitra Pati, Sri Krishna, Aparup Das

**Affiliations:** ^1^Division of Molecular Epidemiology and Public Health, ICMR-Regional Medical Research Centre, Bhubaneswar, Odisha, India; ^2^Division of Vector Borne Diseases, ICMR-National Institute of Research in Tribal Health, Jabalpur, India

## Abstract

Estimation of the spread and advancement of *Plasmodium falciparum* artemisinin-resistant parasites can be done by probing polymorphisms in the kelch (*Pfk13*) domain (a validated molecular marker). This study aimed to provide baseline information for future artemisinin surveillance by analyzing the *k13*-propeller domain in *P. falciparum* field isolates collected from 24 study areas in 14 malaria hot spots of Odisha (previously Orissa) during July 2018-January 2019. A total of 178 *P. falciparum* mono infections were assessed. An 849-base pair fragment encoding the *Pfk13* propeller was amplified by nested polymerase chain reaction and sequenced in both directions (PCR). After DNA alignment with the 3D7 reference sequence, all samples were found to be wild type. It can be anticipated that malaria public health is not under direct threat in Odisha relating to ART resistance.

## 1. Introduction

Malaria infection by *Plasmodium falciparum* is creating a major public health burden all over the world particularly in tropical and subtropical areas. After the clinical failure of Chloroquine and Sulphadoxine–Pyrimethamine due to the occurrence of resistance in South East Asian region and many more countries, Artemisinin or Artemisinin Combination Therapy (ACT) has been recommended by the World Health Organizations (WHO) as the first-line treatment for uncomplicated *P. falciparum* malaria since 2006 [1]. Further continuous reuse of this single ACT resulting in *P. falciparum*-resistant alleles to Artemisinin or its derivatives has been reported for the first time in Western Cambodia and Thailand border [2, 3]; more interestingly, these resistant isolates might be carried by the parasites distributed rapidly across all SE Asian countries and also some parts of Africa [4, 5]. Both Artemisinin Combination Therapies (ACTs) and its derivatives along with partner drug resistance to *P. falciparum* isolates have threatened the current efforts for the reduction of the burden of infectious malaria all over the world [6, 7]

As per the WHO guidelines, the definition of the emerged resistant alleles across Thai and Cambodia border has been characterized by [8, 9] not only with reduced parasite clearance rate and increasing parasite clearance half-life or survived young ring stage parasites but also with the continuation of parasites on the third day of ACTs [1, 10–18]. These worldwide problems give rise to several approaches for the intense surveillance and detection of Artemisinin-resistant *falciparum* parasites including molecular marker evaluation [7, 19]. Genome-wide association studies of the *P. falciparum* whole genome sequencing data have identified a gene named PfKelch13-*PF3D7-1343700* locating on chromosome 13 which encodes K13-propeller protein. The clinical ART failure has been emerged independently by the occurrence of nonsynonymous mutations or polymorphisms in the beta-propeller domain of that kelch protein mutation-expressed substituted AA sequence. But still the question remains unreciprocated why the alleles get transformed into a resistant form and whether they spread geographically to other adjacent countries. In Southeast Asia, Ariey and coworkers [20] have detected some alleles of the K13 propeller genes (C580Y, Y493H, R539T, and I543T) to display delayed parasite clearance. And the rest of the 9 candidate resistance mutations, i.e., P441L, F446I, G449A, N458Y, P553L, R561H, V568G, P574L, and A675V, also were detected by several group of researchers [4, 16, 20–23].

On one hand, in the year 2010, the Government of India launched the National Malaria Control Policy for the complete eradication of malaria infection starting from the rural level by providing LLINs and ACT kits to the village-level health workers ASHA; and the other hand, recent detection of the low frequency, candidate gene mutations of Kelch13 G625R, R539T in certain regions of North Eastern India has put in danger the eradication program. The recent researchers from India could not highlight completely whether these mutant alleles are able to repress the protein expression or causing direct clinical Artemisinin failure [9, 24, 25] remains a research question to be evaluated through molecular genotyping of K13 polymorphism.

The percentage of *Plasmodium falciparum* malaria has been drastically reduced in the year 2018 (81%) as compared with 2012 (93%) in Odisha, which previously remained one of the malaria-endemic state contributing to 40% (until 2017) of the total malaria burden of India [26]. The state geographically lies adjacent to the North-Eastern regions of India. The presence of the above low frequency and nonsynonymous candidate mutation of k13-resistant alleles, whether involved in clinical ART failure in this state or not, could not be ignored in the routine surveillance of *falciparum* malaria for the complete malaria elimination approach in Odisha, India.

## 2. Materials and Methods

### 2.1. Ethical Approval

All the methods of this molecular surveillance study was reviewed and approved by the Institutional Human Ethics Committee of ICMR-RMRC Bhubaneswar, Odisha, India, in accordance with relevant guidelines and regulations of Indian Council of Medical Research (ICMR). The informed consent was obtained from all individual patients prior to blood collection.

### 2.2. Study Area, Sample, and Demographic Data Collection

The study was carried out in both urban and remote areas of the different districts of Odisha, India. 16 sampling sites were selected from localities which were surrounded by dense forest and inaccessible areas, and 9 areas from urban areas as shown in Table 1. The present study was carried out from July 2018 to January 2019. Finger prick bloods of both asymptomatic and symptomatic patients were evaluated for *Plasmodium falciparum* malaria parasites by field-based rapid immunochromatographic test. Patients having Pf/Pv-positive cases were included in this study, and RDT-negative cases were excluded from this study. After obtaining the written informed consent, 1 ml of intravenous blood was collected from the patients ICT positive for Pf/Pv and stored in EDTA vial.

### 2.3. Parasitic DNA Extraction and Genotyping

Parasitic DNA was isolated by using proteinase K and phenol method with slight modifications as described by Sambrook et al.'s molecular cloning and stored at -20°C until use [27].

### 2.4. Nested PCR Amplification

Species-specific nested PCR amplification was carried out to screen the *Plasmodium falciparum* malaria parasites using 18srRNA genes as described in [28, 29]. The *PfKelch13* gene fragment was amplified by nested PCR protocols reported previously by F. Ariey et al. with modifications [20]. The quality and concentration of all the PCR products were analyzed by using 1.5% agarose gel electrophoresis following ethidium bromide stains. The details of nested PCR primers and conditions for the *PfKelch13* conditions are shown in Table 2.

### 2.5. DNA Sequencing

Successful PCR products showing single band in gel were then subjected to PCR purification (using Fast^AP^ alkaline phosphatise and exonuclease I) and further processed for DNA sequencing by Sanger methods (an in-house facility of ICMR-NIRTH, Jabalpur) with 2x coverage (sequenced from both the forward and reverse directions). Briefly, the amplified fragments were sequenced using Big Dye Terminator cycle sequencing ready reaction version 3.1 and ABI Prism DNA sequence Analyzer3130 [17, 20]. For each isolate, sequence chromatograms were viewed carefully, and all the sequences from a single population were aligned (with the help of Gene Doc multiple Sequence Alignment Editor and shading utility version 2.7.000) alongside the reference *Pfkelch13* sequence.

## 3. Results

During the study period, a total of 178 patients (aged 4 to 78 years) were enrolled and diagnosed with *Plasmodium falciparum* by RDT kit and molecular tests shown in Tables 1 and 3. 175 (98.31%) positive cases were detected by RDT and 128 (73.14%) were confirmed *Plasmodium falciparum* through nested PCR diagnosis, by using published primers (rPLU6, rPLU5 primary PCR, and rFAL1 and rFAL2 as nested primer for *falciparum* infections) [29]. 72% (93/128) of the *K13-*propeller gene was amplified by nested PCR method as described in [20] and the thermo cycling conditions shown in Table 2. 57 *PfK13-propeller* genes were successfully sequenced in the selected 64 samples. Samples that could not be amplified were excluded from sequencing; by using this approach, out of 64 monoinfected *P. falciparum* isolates, only 57 (89.96%) could be successfully sequenced for the 849 nucleotide base pair DNA fragment of the *Pfk13* gene. After sequence alignment with the 3D7 reference sequence, all samples were found to be wild type. The nonavailability of novel K13 mutations (neither validated nor candidate) associated with ART resistance to malaria parasites suggested no risk for the Artemisinin Combination Therapy (ACT), in Odisha.

## 4. Discussions and Conclusion

Currently, ACT is the only WHO-recommended antimalarial drug to combat the severity of *Plasmodium falciparum* infection across the globe including Odisha, India. Not only is the emergence and spread of ART-resistant isolate in South East Asian regions a matter of concern but also the occurrence of low-frequency candidate mutations in the k13-propeller gene across certain regions of North-Eastern India could draw the state of Odisha's attention. Since there is no report of transformation of sensitive to resistant alleles reported, molecular evolutionary patter is not known yet in this area of Odisha.

Prior articles from India could not put emphasis on the geographical distribution of validated k13 mutant alleles [9], but the above presented mutations probably create the ACT regimen in high risk for the uncomplicated falciparum malaria in the study areas of Odisha. As Odisha is aiming to reduce malaria particularly *Plasmodium falciparum* infections completely in 2020 to become a malaria-free state, RDT kits and ACTs have been provided to the village-level ASHA workers under the joint action of National Health Mission-National Vector Borne Diseases (NHM-NVBDCP) [26, 27]. Unfortunately, there is no alternative to the current regimen ACT for the severe uncomplicated malaria and also no significant research has yet been done on K13-propeller polymorphism; hence, it is essential to monitor the efficacy of the Artemisinin derivative through molecular surveillance study in the selected areas of the state during the year 2018. Recent study aims to track the research questions raised for the surveillance of falciparum infections by the PCR-based sequencing analysis of Pfkelch13 gene propeller polymorphism.

None of the mutations in the k13 gene whether validated or candidate marker has originated from the current study. Furthermore, the nonoccurrence of resistant alleles in Odisha defines against the contention that the resistant alleles might be distributed in other geographically closer state(s) to Odisha. To the best of our knowledge, this is the first report of a study in Odisha, India, that investigates the efficacy of usual ART resistance through the detection of the Pfkelch13 gene propeller polymorphism study.

Though it may be interpreted from this study that no direct threat to the consequence of ART on the *falciparum* isolates in Odisha, more attention is required on account of the surveillance study for the complete elimination of malaria in Odisha. Field-based survey along with low-cost molecular diagnosis, genotyping of markers related to causing clinical failure of ART should be carried out along with a large number of sample sizes.

## Figures and Tables

**Table 1:** Total number of cases of IDT+ve, nested PCR+ve, *PfKelch13* amplified, and 57 (89.06%) sequenced out of 64 *P. falciparum* isolates, by different sites and districts of Odisha, India.

SL no.	District	SL no. (area wise)	Sites	No. of samples	IDT Pf/Pv	PCR+ve *Pf*	K13 amplified (*n*/%)	Samples sequenced	
*Samples collected from tribal areas (1-16)*									All sequenced *P. falciparum PfKelch13* isolates are of wild type, 89.06% (57 out of 64)
1	Keonjhar	1	Saria	2	2	2	2(100)	1	
2	Kalahandi	2	Banigaon, Lanjigarh	35	35	23	19()	12	
3	Kandhamal	3	Similipadar	31	30	22	18()	13	
4	Kandhamal	4	Saperivatta	8	8	8	6()	3	
5	Sundargarh	5	Sanajala, Baneigarh	1	1	1	1()	1	
6	Sundargarh	6	Uparginia, Baneigarh	15	15	15	5()	4	
7	Angul	7	Rugudidiha	1	1	1	1()	1	
8	Angul	8	Bandhabhui	2	2	2	1()	1	
9	Deogarh	9	Jalisuan, Barkot	5	3	2	1()	1	
10	Raygada	10	Patalamba	12	12	8	7()	4	
11	Raygada	11	Gartali	5	5	5	4()	3	
12	Raygada	12	Kandsui	3	3	3	3()	2	
13	Raygada	13	Parsali	2	2	2	2()	2	
14	Ganjam	14	Uparabartala	5	4	1	1()	1	
15	Malkangiri	15	Andrahal	2	2	2	2()	2	
16	Malkangiri	16	Kanininga	1	1	1	1()	1	
				130	129	107	76(71)	52	
*Samples collected from urban areas CHC, PHC, and dist HQ hospital (17-24)*									
17	Cuttack	17	SCB MCH	21	21	1	1()	1	
18	Khurda	18	Capital Hospital	5	5	4	3()	2	
19	Khurda	19	SUM Hospital	1	1	1	1()	1	
20	Kandhamal	20	RKSSDH, Baliguda	5	2	2	1()	1	
21	Kandhamal	21	Dist HQ hospital, Phulbani	1	1	1	1()	1	
22	Bargarh	22	Paikmal PHC	5	5	4	3()	3	
23	Bargarh	23	Dunguripali, Jharbandh	5	2	2	2()	1	
24	Nuapada	24	Dist HQ hospital, Bukuramunda	5	4	4	3()	2	
				48	46	21	17(80)	12	
			Total	178	175 (98.31%)	128 (73.14%)	93 (72%)	64	

**Table 2:** PCR thermo cycling conditions for primary and secondary PCR (for PF3D7_1343700).

Primer name	PCR	Sequence 5′-3′	Product	Initial denaturation	Denaturation	Annealing	Extension	Final extension	No. of cycles
K13_PCR-F	1^0^ PCR	CGGAGTGACCAAATCTGGGA	2097 bp	95-15 m	95-00.30 s	58-2.00 m	72-2.00 m	72-10.00 m	25
K13_PCR_R	1^0^ PCR	GGGAATCTGGTGGTAACAGC							
K13_N1_F	2^0^ PCR	GCCAAGCTGCCATTCATTTG	849 bp			60-1.00 m	72-2.00 m		28
K13_N1_R	2^0^ PCR	GCCTTGTTGAAAGAAGCAGA							

**Table 3:** Distribution of *P. falciparum* isolates age wise, gender wise, and k13 sequencing results.

Age group	Frequency of Pf cases	%	Pf cases in male	K13 male	Pf cases in female	K13 female		
0-9	36	28.12	27	20	9	5	K13 selected for sequencing no. 64	Propeller sequenced/results (no-wild type, 57 wild type)
10-19	26	20.31	12	8	14	5		
20-29	13	10.15	5	5	8	6		
30-39	9	7.03	4	4	5	4		
40-49	24	18.75	17	12	7	5		
50-59	14	10.93	7	7	7	6		
60-69	5	3.9	2	2	3	3		
70-79	1	0.78	0	0	1	1		
Total	128		74 (57.8%)	58	54 (42.1%)	35		

## Data Availability

The data used to support the findings of this study are available from the corresponding author upon request.
